# Dynamic Adjustment of Weighted GCC-PHAT for Position Estimation in an Ultrasonic Local Positioning System

**DOI:** 10.3390/s21217051

**Published:** 2021-10-24

**Authors:** José Manuel Villadangos, Jesús Ureña, Juan Jesús García-Domínguez, Ana Jiménez-Martín, Álvaro Hernández, Mª Carmen Pérez-Rubio

**Affiliations:** Department of Electronics, University of Alcalá, 28801 Madrid, Spain; jm.villadangos@uah.es (J.M.V.); jjesus.garcia@uah.es (J.J.G.-D.); ana.jimenez@uah.es (A.J.-M.); alvaro.hernandez@uah.es (Á.H.); mcarmen.perezr@uah.es (M.C.P.-R.)

**Keywords:** generalized cross-correlation, ultrasonic local positioning system, multilateration

## Abstract

Ultrasonic local positioning systems (ULPS) have been brought to the attention of researchers as one of the possibilities that can be used for indoor localization. Acoustic systems combine a suitable trade-off between precision, ease of development, and cost. This work proposes a method for measuring the time of arrival of encoded emissions from a set of ultrasonic beacons, which are used to implement an accurate ULPS. This method uses the generalized cross-correlation technique with PHAT filter and weighting factor *β* (GCC-PHAT-*β*). To improve the performance of the GCC-PHAT-*β* in encoded emission detection, the employment is proposed of mixed-medium multiple-access techniques, based on code division and time division multiplexing of beacon emissions (CDMA and TDMA respectively), and to dynamically adjust the PHAT filter weighting factor. The receiver position is obtained by hyperbolic multilateration from the time differences of arrival (TDoA) between a reference beacon and the rest, thus avoiding the need for receiver synchronization. The results show how the dynamic adaptation of the weighting factor significantly reduces positioning errors from 20 cm to 2 cm in 80% of measurements. The simulated and real experiments prove that the proposed algorithms improve the performance of the ULPS in situations with lower signal-to-noise ratios (SNR) than 0 dB and in environments where the multipath effect makes it difficult to correctly detect the encoded ultrasonic emissions.

## 1. Introduction

Technology dedicated to determining the position of an object, robot, or person, either in an outdoor or indoor space (building, premises, or room) has experienced a rapid growth during the last decade. Currently there is a wide variety of fields where location technology can be applied, such as real-time inventory control, merchandise tracking, mobile robotics, motion capture applied to virtual reality, video games, security systems, people routine assessment, etc. State-of-the-art systems can determine position with an accuracy from a few millimeters to hundreds of meters. However, for indoor applications, these systems are strongly dependent on the technology used and the actual working environment, making it a field still in fervent development.

The development of ambient intelligence in recent years has been very relevant and is clearly having a great impact on improving people’s lives. Its interdisciplinary nature is leading to different areas of expertise coming together for a common purpose. The ultimate goal of ambient intelligence is the creation of habitable spaces (also called intelligent environments) in which users interact naturally with computational services that facilitate the performance of their daily tasks, whether for leisure or work, or even health care both in hospitals and at home. Its foundations go back to the concept of ubiquitous computing proposed by Weiser [1] and have been fed by the results of many other research areas, such as communications and mobile devices, multimodal user interfaces, artificial agent systems, sensors, computational vision, and home automation, among others. Ambient intelligence implies that through sensory technology, consumer electronic devices can recognize the context and behave according to (context-aware) who is using them [2], or where, when and how they are being used. Although the fields of application can be very diverse, what is common to all of them is the need for a local positioning system (LPS) that allows the obtaining of the position of the mobile elements of the environment (robots, people, etc.) in a seamless way.

Practical indoor positioning systems rely on data acquired from mobile or onboard sensors, which can be affected by fluctuations caused by the environment. This source of data may provoke cumulative errors that must be removed [3]. Relative positioning-based techniques include inertial navigation systems (INSs) [4], light detection and ranging (LiDAR) [5], visual simultaneous localization and mapping (SLAM) using cameras [6], and pedestrian dead reckoning (PDR) [7]. Different approaches attempt to solve the problem of cumulative error in relative systems using additional sensors based on primary technologies, as radio frequency (RF), optics, acoustics, etc. 

The most used technology is RF, since many solutions are based on the use of stablished networks. This is the case of the WiFi infrastructure [8], which can reach accuracies of several meters after a calibration process (fingerprinting), with the problem that is necessary to perform a new calibration whenever several conditions change (furniture distribution, WiFi access points, etc.). On the other hand, multi-antenna approaches can achieve centimetric accuracy [9]. Ultra-Wide Band (UWB) technologies have emerged as a suitable solution for indoor positioning, even included in current smartphones, providing accuracies below decimeters [10,11]. Additionally, 5G networking makes use of its rich bandwidth and the processing and communications capabilities. The work in [12] makes a survey of the opportunities and challenges of 5G positioning. 

Optical signals, which can be infrared or visible light, typically employ LEDs as transmitters and imaging sensors or photodetectors as receivers. For instance, when using coding techniques with LED emissions and Quadrature Angular Diversity Aperture (QADA) receivers, positioning results with precision in the range of a few decimeters can be obtained [13]. This performance is similar to the one achieved with acoustic systems [14], on which this work focuses. Both acoustic and optical systems are constrained by the physical limits of the indoor spaces (walls, furniture, etc.). 

The use of beacons is a well-known technique in LPSs, and, for the cases in which a centimeter accuracy is needed, UWB and ultrasound are the most used technologies [15,16,17]. The signal fluctuations caused by the structure of the building is one of the main difficulties to deal with, as the signals from the beacons can follow complex paths, i.e., non-line-of sight (N-LOS) propagation [18]. The LPS can be structured following two types of strategies [19]. On one side, if the beacons are receivers and the tag to be positioned incorporates the transmitter, it constitutes a centralized system that requires the beacons to be in communication with each other or with a central system to obtain the absolute position of the transmitter. If several transmitters are to be located simultaneously, i.e., several robots or people, it is necessary to incorporate some multiple-access media techniques or to establish some kind of synchronization with the beacons, requiring more processing time to obtain the position of each transmitter. On the other hand, if the beacons are transmitters, the receiver estimates its position (self-positioning), thus being a decentralized system characterized by its privacy. In this second case, the position calculation time is independent of the number of devices to localize.

### Use of Coded Emissions and Cross-Correlation Techniques 

In those LPS based on a group of transmitter beacons located at known positions, which transmit simultaneously and periodically, the DS-CDMA (Direct-Sequence Code Division Multiple-Access) technique is often considered to be a feasible approach to avoid any crosstalk interference [14,16]. The mobile object estimates its positioning by detecting the beacons’ emissions. In this process, each mobile object measures the time of arrival (ToA) or the time differences of arrival (TDoA) to determine the distance or distance difference between each mobile node and the different beacons. As the emissions are encoded, the first approach to detect their arrival in the receiver is using the cross-correlation as a matched filter, with suitable binary sequences encoding the emissions, and followed by different processing techniques at the receiver to make the system more robust against multipath, low coverage areas, near-far effect or Doppler effect [20,21].

The TDoAs are determined by the peak detection of the cross-correlation function, with the advantage of relative fast calculation and good robustness. Nevertheless, fuzzy main peaks or multiple peaks appear under low SNR or other harmful effects (multipath, bandwidth limitations, …). To sharpen the peak of the cross-correlation function, one of the methods used is the generalized cross-correlation (GCC) method, which increases the peak through a pre-whitening process [22].

The authors in [23] proposed the use of generalized cross-correlation with PHAse Transform filtering (GCC-PHAT) for the detection of ultrasonic encoded emissions, showing an improvement in the TDoA estimations and, consequently, in the final positioning. The main reason of this improvement is that GCC-PHAT provides a significant reduction in the sidelobe effects (when compared with the standard correlation). The origin of the GCC-PHAT lies in the proposal from [24] to determine the delay times of acoustic signals received at various microphones. An example of recent improvements includes the combination of the GCC and the phase transform in the wavelet domain [25]. Additionally, many authors have proposed the introduction of a weighting factor (*β*) in the PHAT filter (PHAT-*β*), to improve such estimation with acoustic signals [26]. Generally, this weighting factor is empirically fixed, depending on the environment and the type of signals used. Although it requires some previous experiments, the weighting factor has been beneficial for improving the detection in environments with a certain degree of reverberation [27,28]. 

This work presents an ultrasonic LPS (ULPS) based on an encoding scheme for the beacon emissions with a mixed multiple medium access technique, which consists of a code division and time multiplexing of the beacon transmissions (CDMA and TDMA). With this mixed scheme (T-CDMA), the estimation of the ToA of the emissions is improved regarding the use of a generic CDMA scheme, and without the time penalization related to a common TDMA method. The proposal for processing the received signal is the use of a GCC-PHAT-*β* filter to adjust dynamically the weighting factor *β*. The weighting factor is obtained from the signal received in each calculation window, which reflects the environmental conditions during this interval of transmission time. A large set of simulated and real experiments proves that the proposed algorithms improve the performance of the ULPS in situations with lower SNR than 0 dB and in environments where the multipath effect makes it difficult to correctly detect the arrival of the encoded ultrasonic emissions with other traditional methods. 

This paper is organized as follows: Section 2 describes the ULPS structure developed, the system model proposed, and how the measurement of TDoAs is carried out using the GCC-PHAT; Section 3 deals with the dynamic adjustment of the weighted GCC-PHAT-*β*; Section 4 provides some simulated and real positioning results; and finally, conclusions are discussed in Section 5.

## 2. Description of the Ultrasonic Local Positioning System

### 2.1. ULPS Structure

**Figure 1:**
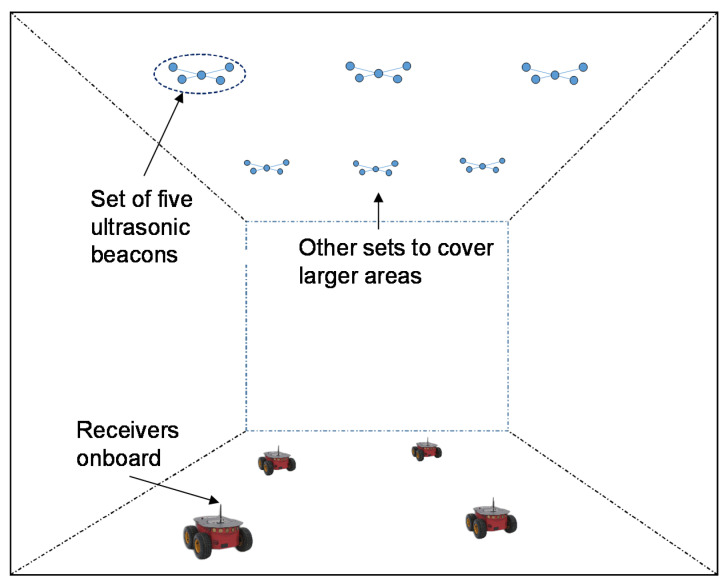
Deployment of ULPS for a wide coverage area using sets of five ultrasonic beacons.

Figure 1 depicts the structure of the LOCATE-US positioning system developed by the GEINTRA group from the University of Alcalá [14,16], involved hereinafter.

**Figure 2:**
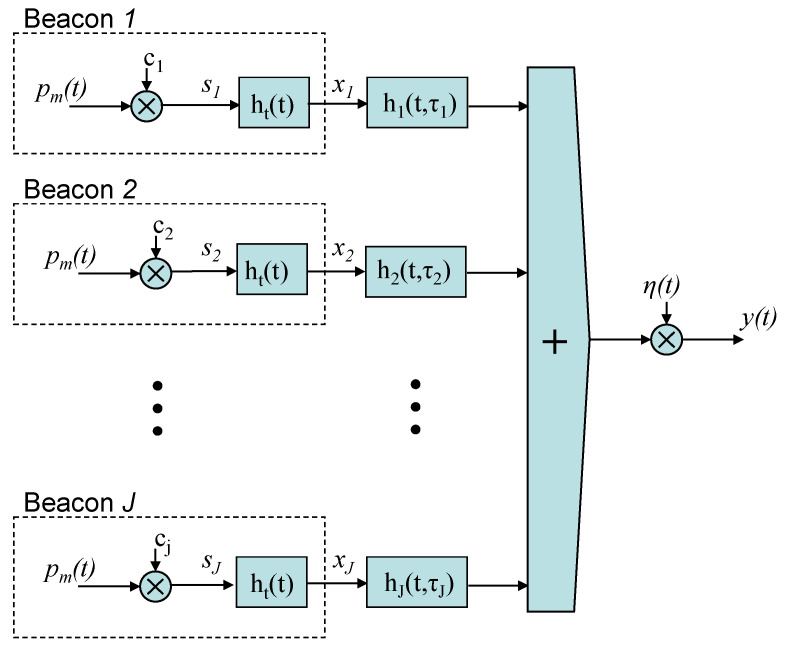
Propagation model in an ULPS with *J* emitters.

The model of the positioning system proposed can be observed in Figure 2, for every set of *J* emitters. Every emitted signal *x_j_*(*t*) is the encoded emission of the beacon *j*, coming from the modulation of a binary code *c_j_* with a carrier *p_m_*(*t*) (generating a sequence *s_j_*(*t*)), and the convolution of this modulated signal with the impulse response of the transmitter, *h_t_*(*t*). Considering that *h_j_*(*t,τ_j_*) is the channel response for the beacon *j*, *τ_j_* is the delay of the sequence assigned to beacon *j* at the receiver, and *η(t)* is a zero-mean Gaussian noise with variance *σ^2^*, the received signal *y*(*t*) is given by (1):

(1) $$y\left(t\right)=\overset{J}{\underset{j=1}{\sum }}x_{j}\left(t\right)*h_{j}\left(t,\tau_{j}\right)+\eta \left(t\right)$$

**Figure 3:**
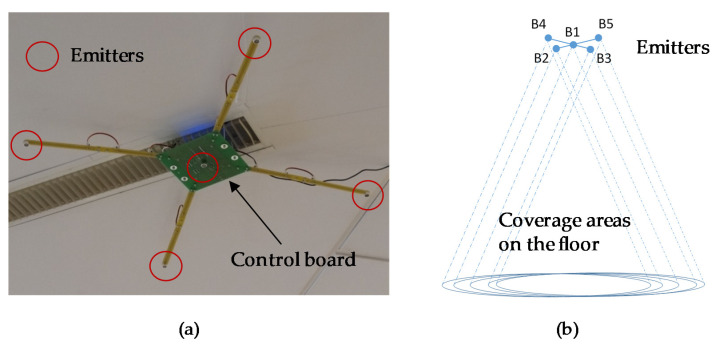
(**a**) Detail of the real prototype. (**b**) Common coverage area for one set of five beacons.

The minimum ULPS is based on one set of five beacons located at known positions that transmit simultaneously and periodically using DS-CDMA and TDMA (T-CDMA) techniques. Figure 3a) shows the distribution of the five beacons: four of them form a square with a side of 70 cm and the fifth one is at the center. This distribution, with the beacons so close one each other, penalizes the PDOP (Precision Dilution of Position) in the common coverage area (Figure 3b). On the contrary, this distribution has advantages to minimize the impact of other well-known problems, such as the *near-far* effect (if only the nearest beacons are detected at the receiver), since all the beacons have a similar emitted power in the common coverage area. Each set of five beacons has a coverage area of about 50 m^2^ on the floor, when the height of the ceiling is 3.5 m and the aperture angle of the emitters is 120° (in the receiver this angle can be close to 180°). This coverage can be enlarged including in the room more sets of beacons (remember the representation in Figure 1), emitting pseudo-orthogonal codes to avoid interferences (for instance, with Kasami codes of 1023 bits, there are up to 32 pseudo-orthogonal codes). Additionally, the sets of ULSPs with five beacons can be placed with enough distance between them, as, in most applications, a high precision is only required in particular areas, whereas positioning in the rest of the indoor space can be achieved with relative positioning-based techniques (with onboard sensors) that reset their cumulative errors when entering in a ULPS covered area.

**Figure 4:**
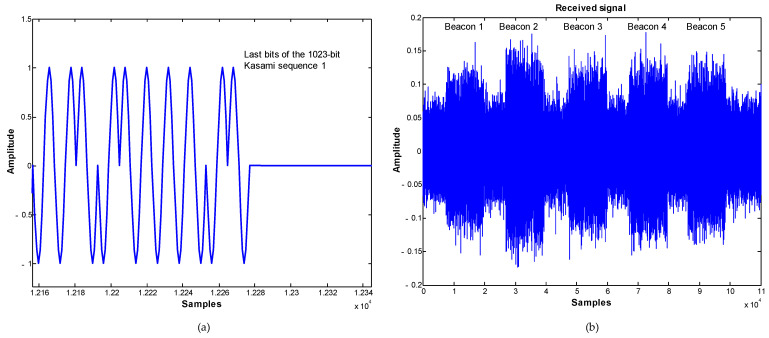
(**a**) BPSK-modulated carrier of a Kasami sequence with 1023 bits (last bits). (**b**) Received signal corresponding to the five beacons-multiplexed emissions in a noisy channel.

Each beacon emits a BPSK-modulated 1023-bit Kasami code with a 41.667 kHz sine carrier. The modulation symbol is composed of one or several carrier cycles to adapt the signal to the bandwidth of the emitter and to provide the necessary energy to the channel. In the proposed system the symbol is formed of 12 samples per carrier cycle, thus implying a sampling frequency of 500 kHz [29]. Figure 4a) shows the last bits of a 1023-bit Kasami sequence, and Figure 4b) the reception of the five beacons-multiplexed emissions with additive Gaussian noise. 

Please note that this LPS does not need synchronization between the beacons and the receiver, as the positioning algorithm is based on hyperbolic trilateration. In this case, the TDoAs between a reference beacon and the others are measured. To do that, at the receiver, the signal is acquired and sent to a buffer with enough capacity to store one complete transmission period (a window of approximately 200 ms).

### 2.2. TDoA Determination

**Figure 5:**
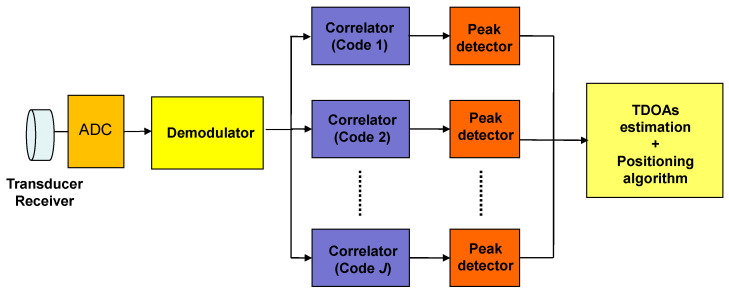
Block diagram of the receiver (ADC stands for Analog to Digital Converter).

The arrival of each transmitter’s emission is obtained by the correlation between the received signal *y*[*n*] and the corresponding transmitted code *c_j_*, by detecting when a maximum correlation peak appears. Figure 5 shows a receiver block diagram. 

Due to the non-idealities of the auto-correlation (AC) and cross-correlation (CC) functions of the sequences used and the influence of the transmission channel, Inter-Symbol Interference (ISI) and Multiple-Access Interference (MAI) may appear. The effect of these interferences is that the correlation peak is not ideal, appearing sidelobes that make it difficult the detection of the real main peak position, required for determining the ToA. Algorithms for MAI and ISI cancellation are used typically in communications, which usually involve a high computational load. In this work, to improve the detection process, we propose the use of generalized cross-correlation (GCC) [23], which is widely employed in acoustic systems to estimate the delay time between a signal received by different microphones [24].

The GCC in the discrete domain between the received signal *y*[*n*] and the sequence *s_j_*[*n*] to be detected, named $\varphi_{s_{j}}^{GCC}$, for a signal-length of *K* samples, is given by (2):

(2) $$\varphi_{s_{j}}^{GCC}\left[n\right]=\overset{K-1}{\underset{k=0}{\sum }}\Phi_{j}\left[k\right]\cdot S_{j}\left[k\right]\cdot Y^{*}\left[k\right]\cdot e^{ik\frac{2\pi }{K}n}$$

where Y^*^[*k*] is the conjugate of the discrete *Fourier* transform of the received signal *y*[*n*]; $\Phi_{j}\left[k\right]\;\mathrm{and}\;Sj\left[k\right]$ are the discrete *Fourier* transforms of the weight function and the sequence *s_j_*[*n*] to be detected, respectively. 

The weight function represents a previous filtering of the received signal and the sequence to be detected, with the aim of emphasizing the peak of the cross-correlation function. This filter is equivalent to applying a weight function to the cross-spectral density function between the received signal and the sequence to be detected. If *G_Sj,y_*[*k*] represents the cross-spectral density function between *s_j_*[*n*] and *y*[*n*], then Equation (2) can be expressed as (3):

(3) $$\varphi_{s_{j}}^{GCC}\left[n\right]=\overset{K-1}{\underset{k=0}{\sum }}\Phi_{j}\left[k\right]\cdot G_{s_{j},y}\left[k\right]\cdot e^{ik\frac{2\pi }{K}n}$$

It is worth noting that if $\Phi_{j}\left[k\right]=1$ the GCC function in (3) becomes the standard cross-correlation (CC). The estimated time instant $n={\hat{D}}_{j}$ corresponds to the maximum of the function in (3), and represents the arrival of the sequence *s_j_*[*n*] at the receiver, which is defined by (4):

(4) $${\hat{D}}_{j}=arg\;\underset{n}{max}\varphi_{s_{j}}^{GCC}\left[n\right]$$

The TDoA $\tau_{ij}\;$between a reference beacon *i* and another beacon *j*, considering the sampling time *T_s_*, is given by (5):

(5) $$\tau_{ij}=T_{s}\cdot \left({\hat{D}}_{i}-{\hat{D}}_{j}\right)=T_{s}\cdot \left(arg\;\underset{n}{max}\varphi_{s_{i}}^{GCC}\left[n\right]-arg\;\underset{n}{max}\varphi_{s_{j}}^{GCC}\left[n\right]\right)$$

The filtering function known as PHA Transform (PHAT filtering) is widely used to estimate the delay of a signal that arrive to two receivers located at a given distance from the acoustic source. Considering the beacons as emitters, each one with an emitted sequence *s_j_*[*n*], and the signal *y*[*n*] at the receiver, the filter expression $\Phi_{j}\left[k\right]$ is as follows:

(6) $$\Phi_{j}\left[k\right]=\frac{1}{\left|S_{j}\left[k\right]\cdot Y^{*}\left[k\right]\right|}$$

The estimation of the time of arrival for each beacon, when applying the GCC with PHAT filtering, requires a more complex analysis than the standard cross-correlation. This is because the received signal is a composition of as many “signals” as available beacons. More detail can be found in the previous work presented in [23].

## 3. GCC with Weighting Factor

Sometimes neither the standard cross-correlation nor the GCC-PHAT provide suitable results. However, the performance of the GCC with PHAT filter can be improved by including a weighting factor in the PHAT filter [26]. The proposal is to modify the modulus of the power cross-spectrum between the received signal and the sequence according to a parameter or factor *β* (0 ≤ *β* ≤ 1). Then, Equation (6) can be re-written as follows:

(7) $$\Phi_{j}^{’}\left[k\right]=\frac{1}{{\left|S_{j}\left[k\right]\cdot Y^{*}\left[k\right]\right|}^{\beta }}$$

In this case, the GCC-PHAT correlation with weighting factor *β* ($R_{y,s_{j}}\left[n\right]$), denoted as GCC-PHAT(*β*) hereinafter, between the received signal *y*[*n*] and the sequence to be detected *s_j_*[*n*], is given by (8). Please note that if *β* = 0, the expression corresponds to the standard correlation, and, if *β* = 1, it does to the generic GCC-PHAT.

(8) $$R_{y,s_{j}}\left[n\right]=\frac{1}{K}\overset{K-1}{\underset{k=0}{\sum }}\frac{1}{{\left|S_{j}\left[k\right]\cdot Y^{*}\left[k\right]\right|}^{\beta }}\cdot S_{j}\left[k\right]\cdot Y^{*}\left[k\right]\cdot e^{i\frac{2\pi k}{K}n}$$

The value of the weighting factor *β* in the GCC-PHAT has a great influence on the correlation result. When the SNR ratio worsens, the PHAT filter must be less demanding on the phase response of the power cross-spectrum between the received signal *y*[*n*] and the sequence *s_j_*[*n*], to guarantee a better detection of the maximum of the correlation function. As can be observed afterwards in Section 4, it has been empirically proven that with this particular system, for lower SNR values than 0 dB it is necessary that *β* is below 1, and this variation is related to the SNR at the receiver.

### Dynamic Adjustment of Weighted GCC-PHAT

As indicated above, there is a great influence of the weighting factor *β* on the final correlation result, according to the SNR in the working environment. Therefore, we propose to dynamically adjust the weighting factor so that it adapts to the operating conditions in which the ULPS is working. 

Another advantage of using the T-CDMA technique, in addition to the improvement of the ToA estimation regarding the use of only CDMA, is the possibility of evaluating at the receiver the secondary peak to main peak ratio (SPMP ratio) in the signal coming from each beacon independently, since there is no overlapping between the different emissions. The proposal is to find a relationship between the weighting factor *β* and the SPMP to dynamically adapt the value of *β* to improve the GCC-PHAT performance. Then, after each correlation, the SPMP can be computed, by detecting the maximum value of the correlation and the value of the sidelobes. Through this SPMP, we can determine the most optimal value of *β* for each sequence independently, apply that factor in the next detection, and thus decrease the estimation error of the mobile position within the LPS coverage area, since a wrong detection of the time instant of the sequence arrival results in a positioning error. 

**Figure 6:**
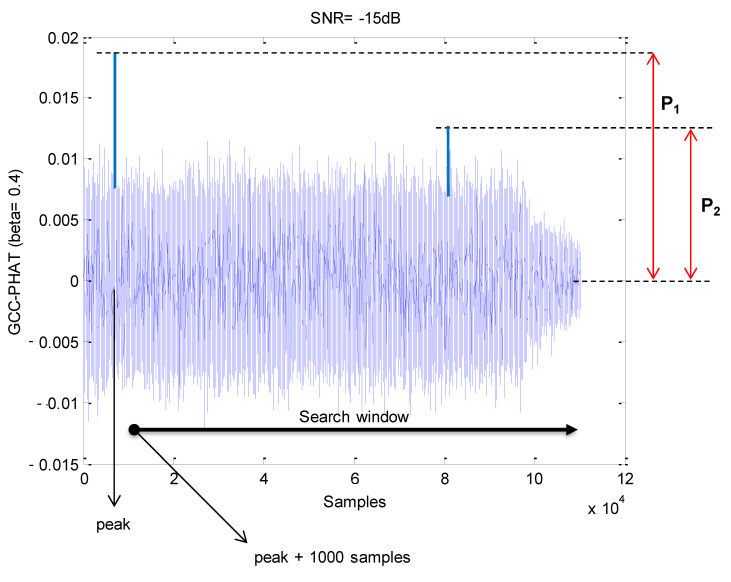
Example of detection using the GCC-PHAT for a beacon (SNR = −15 dB).

Figure 6 shows an example of the GCC-PHAT between the received signal and the sequence corresponding to a beacon with simulated data. Please note that with the SNR = −15 dB selected, the main peak has an approximately double amplitude than the average of noise around, and thus it can be detected. The SPMP is estimated from the ratio *P* between the maximum *P*_2_ of the sidelobes outside the multipath zone (first 1000 samples around the main peak) and the main lobe *P*_1_ (*P* = *P*_2_/*P*_1_). Please note that the lower the value of P the better the SPMP ratio, including process noise.

It is required a model that determines the behavior of *β* as a function of SPMP. We have carried out an empirical analysis for different SNR values. To simplify the model, we have considered that the multipath effect only affects the central beacon of the ULPS (beacon 1), according to the model described in Section 2. The proposal consists of considering a set of fixed SNRs, and varying *β* to analyze the values of SPMP, i.e., the values of *P*, for each SNR. The aim is to obtain an optimal value of *β* that minimizes the factor *P*, in such a way it guarantees the best behavior of the GCC-PHAT(*β*) to obtain the maximum of the correlation function. 

**Figure 7:**
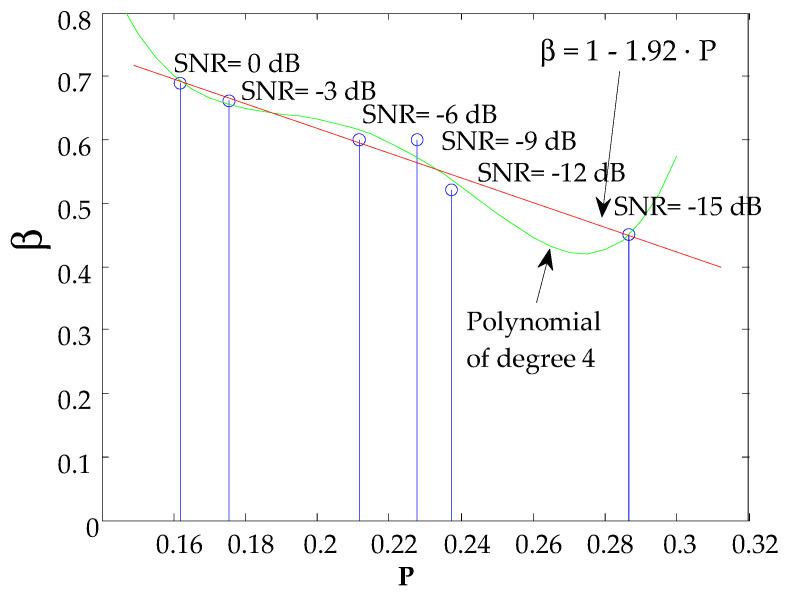
Estimation of the *β*-factor in the GCC-PHAT, as a function of the SNR (SNR < 0 dB) and the factor *P*.

A range of SNR variation [−15 dB, −12 dB, −9 dB, −6 dB, −3 dB, 0 dB] has been considered in the simulated test. The reason to select these values is that in this range the value of *β* has influence: lower SNR can end up with problems to detect the main correlation peak and higher SNR can be computed with the non-pondered GCC, i.e., *β* = 1, as is shown further on. For each SNR, *β* has been modified between 0.4 and 1 in steps of 0.01, obtaining the value of *β* for which P is minimum. Figure 7 shows the results extracted from the test. It is represented in green a 4th order polynomial that best models the behavior of *β*. However, we have considered the linear approximation function (straight line in red) expressed in (9), due to its low computational cost and the fact that the differences of *β* using one or another approximation is typically much less than 0.05, what would have a negligible impact in real situations, where there can be other sources of noise (not only Gaussian), the model of multipath can be different, etc.:

(9) β=1−1.92·P

## 4. Positioning Results

### 4.1. General Considerations

**Figure 8:**
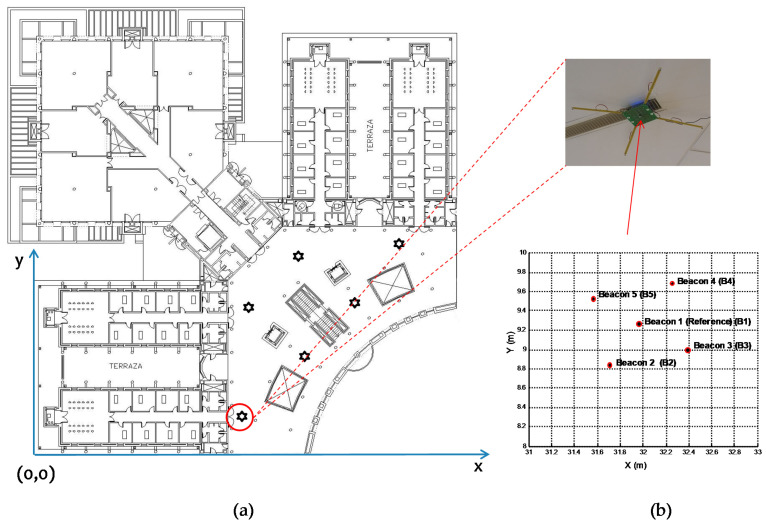
(**a**) Experimental placement of the ULPS to consider the global coordinates of the building map (the rest of ULPSs not marked have not been used in this experiment); (**b**) projections of the beacons on the floor plan (*z* = 0).

As previously described, the beaconing system considered consists of five beacons, four at the corners of a 70 cm-side square and the fifth one at its center (as is shown in Figure 3), located at a height of approximately 3.5 m above the ground. Figure 8a) shows the location of the beaconing system in the building where the tests were performed, as well as the origin of coordinates. Figure 8b) shows the projection of the beacons on the ground plane (*z* = 0). A global reference system given by the geometry of the building has been considered. The coordinates of the beacons have been obtained from the calibration system proposed in [30].

Simulation tests have been carried out considering the transducer model, bandwidth, emission pattern, and the impulse response of the channel modeling the multipath effect. Some parameters, such as the signal propagation velocity and the standard deviation of the ToA error (2 µs), have been considered constant for all the simulation tests.

**Figure 9:**
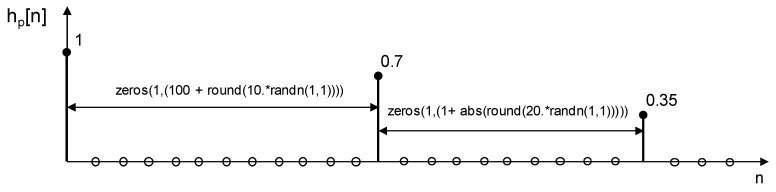
Channel impulse response that models the multipath effect considered for simulations.

To model the multipath effect, we have assumed that the channel has an impulse response as shown in Figure 9, simulating two paths with attenuations of 0.7 and 0.35 respectively, with some degree of randomness for time delay. The choice of this simple model allows simulation of the effect of a typical multipath found when the receiver is close to a wall and other piece of furniture. The received signal is the addition of the LOS signal (assumed with a reference ponderation of 100%), other signal with an amplitude ponderation of 70% and delayed a random value of samples between 101 and 110, and another with an amplitude ponderation of 35% and delayed, regarding the previous one, a random value of samples between 1 and 21. 

To obtain the position of a receiver within the coverage area of the developed ULPS, the following process was followed: (1) the time instant of arrival of the signal from every beacon was obtained by applying both the cross-correlation (CC) and the GCC-PHAT(*β*) (the position of the maximum value of the correlation was considered to be the time instant of arrival); (2) the final receiver’s position was estimated by hyperbolic trilateration through the Gauss-Newton method [31], using as reference the central beacon (beacon 1), so the TDoAs are defined as $\tau_{1j}$, *j* = {2, 3, 4 and 5}.

To evaluate how the weighting factor affects the positioning error, we first introduce a series of results corresponding to the simulation of the indicated ULPS, using 1023-bit Kasami sequences and taking 50 measurements at various test points on the ground surface. In all the cases under study, the statistical error analysis is carried out without considering the outliers that may appear, especially in situations with low SNR. The filtering criterion for outliers, assuming a normal distribution with zero mean and deviation σ, has been 3·σ around the mean value, when evaluating the position error for the *X* and *Y* coordinate axes (2D). Secondly, we have analyzed the position error of a mobile robot on a simulated circular trajectory on the ground plane. Finally, experimental results are provided for the same mobile receiver and trajectory, implementing the aforementioned algorithms for the ULPS. 

### 4.2. Effect of the Weighting Factor

**Figure 10:**
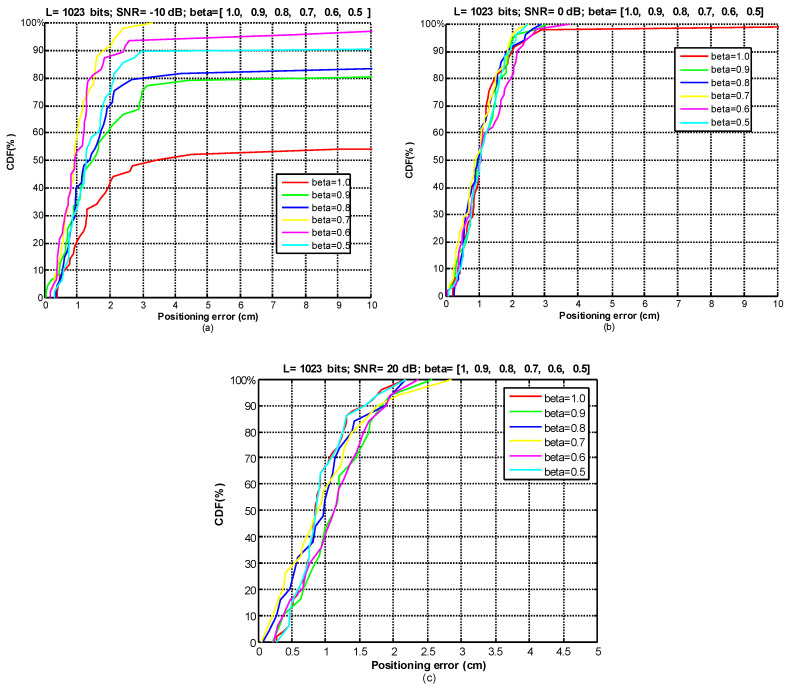
CDFs of ranging errors in 2D positioning applying the GCC-PHAT(*β*) at a far point. (**a**) SNR = −10 dB; (**b**) SNR = 0 dB; (**c**) SNR = 20 dB.

For the analysis of the weighting factor *β* we have used a set of SNR values (−10 dB, 0 dB, 20 dB) and we have varied *β* between 0.5 and 1 to evaluate its effect on the positioning error. For that purpose, we have analyzed the positioning results (50 measurements) with the receiver at a far test point (around 6 m from the center), and we have obtained the Cumulative Distribution Function (CDF) of ranging errors in 2D positioning between each estimated position and the ground-truth position of the test point. As Figure 10a) and Table 1 show, for SNR = −10 dB, the best results (in bold) are obtained when *β* = 0.7, reducing the positioning error to 2 cm for 90% of measurements and with only 2% of outliers. The error considerably increases as *β* approaches 1 (note that *β* = 1 corresponds to non-pondered GCC-PHAT), increasing the number of outliers up to 30% of measurements. However, as the SNR increases, we observe that the weighting factor has a lower influence. For the case of SNR = 0 dB (Figure 10b), for most of the *β* values the error is around 2 cm for 90% of measurements, and for SNR = 20 dB (Figure 10c) the error is lower than 2 cm for more than 90% of cases. 

**Figure 11:**
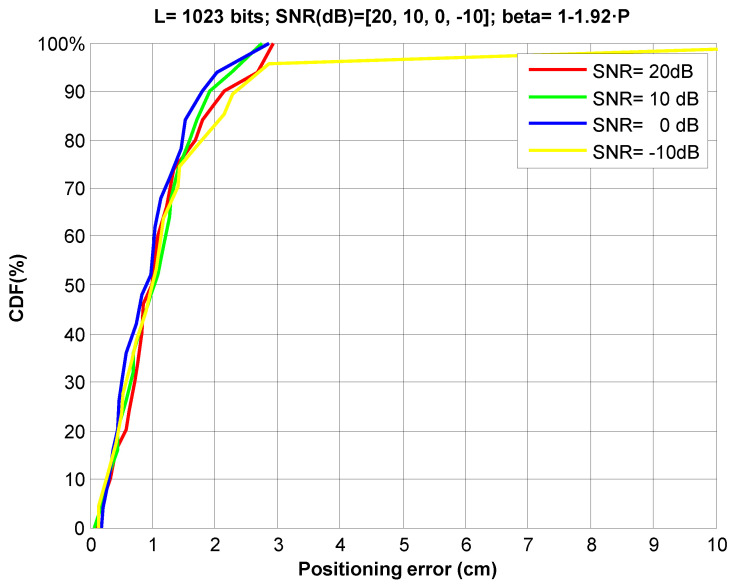
CDF of ranging errors in 2D positioning applying GCC-PHAT(*β*) at a far point, for different SNRs at the receiver, with the proposed dynamic estimation of the weighting factor (*β* = 1 – 1.92·*P*).

Then, we have simulated the dynamic estimation of the PHAT filter weighting factor given by (9Figure 11 depicts the CDF of positioning errors for several SNR values (−10 dB to 20 dB). As the results show, the weighting factor is adequately adjusted, keeping the ranging error around 2 cm for 90% of measurements, cancelling the negative effect from low SNRs on the positioning.

### 4.3. Simulation Results for a Moving Receiver

As the benefits of using GCC-PHAT(*β*) when the receiver is at a fixed position have already been described, the following subsection focuses on evaluating it when the receiver is continuously moving on a plane on the ground (coordinate *z* = 0). We have simulated a receiver following a circular path of 3 m radius, whose center is located at the projection on the plane *z* = 0 of beacon 1 (the reference beacon). We have divided the path into four equal parts of 90° (Q1-Q4), and for each one we have used a different SNR, with and without multipath effect. The features of each quadrant are as follows: Q1 (0–90°), SNR = −10 dB (without multipath); Q2 (90°–180°), SNR = 10 dB and multipath effect; Q3 (180°–270°), SNR = 0 dB and multipath effect; and Q4 (270°–360°), SNR = −10 dB and multipath effect. For analyzing the effect of the proposed GCC-PHAT(*β*), we compare the results with the CC. A set of 360 points has been evaluated along the path. 

**Figure 12:**
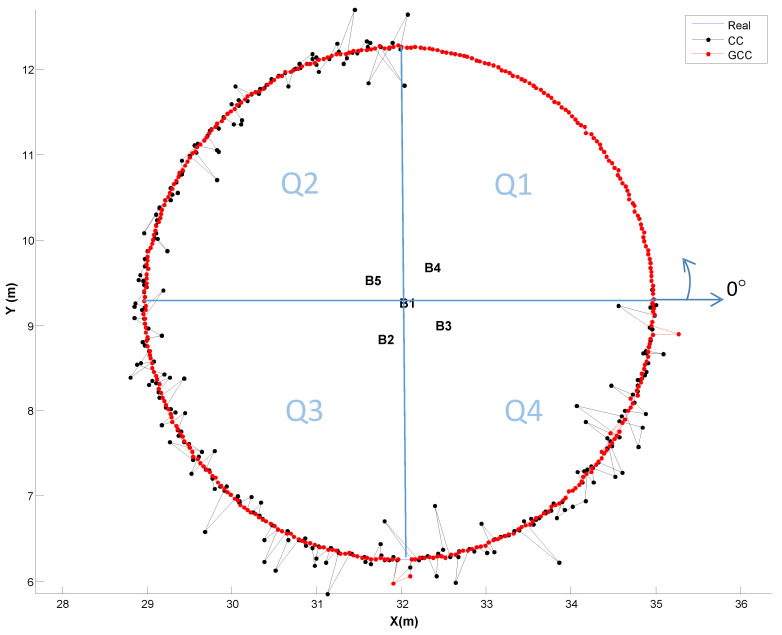
2D positioning estimation on a circular path of 3 m radius. Different SNR conditions have been simulated per quadrant: Q1 (0–90°), SNR = −10 dB (without multipath); Q2 (90°–180°), SNR = 10 dB and multipath effect; Q3 (180°–270°), SNR = 0 dB and multipath effect; and Q4 (270°–360°), SNR = −10 dB and multipath effect.

**Figure 13:**
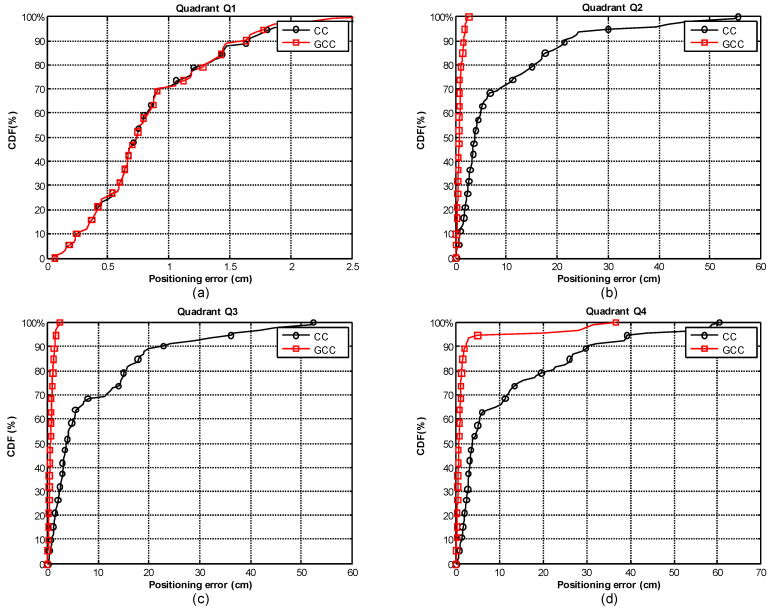
CDF of ranging errors in 2D positioning when applying the CC (black) and the GCC-PHAT (red) with dynamic *β* estimation for the path shown in Figure 12: (**a**) quadrant Q1 (0–90°), SNR = −10 dB (without multipath); (**b**) quadrant Q2 (90°–180°), SNR = 10 dB and multipath effect; (**c**) quadrant Q3 (180°–270°), SNR = 0 dB and multipath effect; (**d**) quadrant Q4 (270°–360°), SNR = −10 dB and multipath effect.

Figure 12 and Figure 13 show the obtained positioning results when using the CC or the dynamic adjustment of the weighting factor *β* for the GCC-PHAT filter, estimated at each time instant and applied to the next measurement (as was described in Section 3). 

Figure 12 shows in the first quadrant Q1, where in the absence of multipath, the behavior of GCC-PHAT(*β*) (in red) versus CC (in black) is very similar despite the low SNR (−10 dB), resulting in an error lower than 1.5 cm, with a confidence of 90% (Figure 13a). However, with the presence of multipath effect (quadrants Q2, Q3), we can visually appreciate in Figure 12 that the performance of GCC-PHAT(*β*) is better than CC. 

In the second and third quadrants (Q2, Q3) with SNR of 10 dB and 0 dB respectively, GCC-PHAT(*β*) achieves a constant error lower than 2 cm, whereas CC provides errors up to 20 cm (Figure 13b,c, respectively). 

In the fourth quadrant Q4 and with a very low SNR = −10 dB, the GCC-PHAT(*β*) keeps an error lower than 3 cm, whereas the CC reaches errors of up to 30 cm (Figure 13d). If we reduce the confidence factor to 80%, the position error obtained by the GCC-PHAT(*β*) remains below 2 cm, whereas it is up to 20 cm with the CC. 

The above results clearly show that the use of the GCC-PHAT(*β*), with a dynamic adjustment of the weighting factor, significantly improves the detection of the maximum correlation value, thus improving the measurement of TDoAs, and therefore decreasing the positioning error. This improvement is noticeable when the SNR is low, and the multipath effect exists (ordinary working conditions with ULPSs). 

Please note that with higher SNR than 0 dB the value of *β* can be set at 1, thus meaning that the GCC-PHAT (without ponderation) works properly. For interested readers, this situation was analyzed in [23]. Assuming that the noise is completely uncorrelated, peaks in the GCC are much narrower than in CC, close to a true delta function without sidelobes. The applied PHAT filtering enhances the real instant of arrival of the sequence and eliminates spurious delays, to a greater or lesser extent, depending on noise power. The advantage of PHAT over other filtering functions is that it notably improves the arrival estimation in environments with a certain level of reverberation. When the SNR is lower than 0 dB, the level of noise negatively affects the PHAT filter and, then, values of *β* below 1 provide better results. As *β* tends to 0, the GCC tends to be a standard CC, so it is possible to state that with the value of *β*, the cross-correlation is adjusted between the GCC (*β* = 1) and the standard CC (*β* = 0), depending on the SNR. 

### 4.4. Experimental Results with a Mobile Receiver

**Figure 14:**
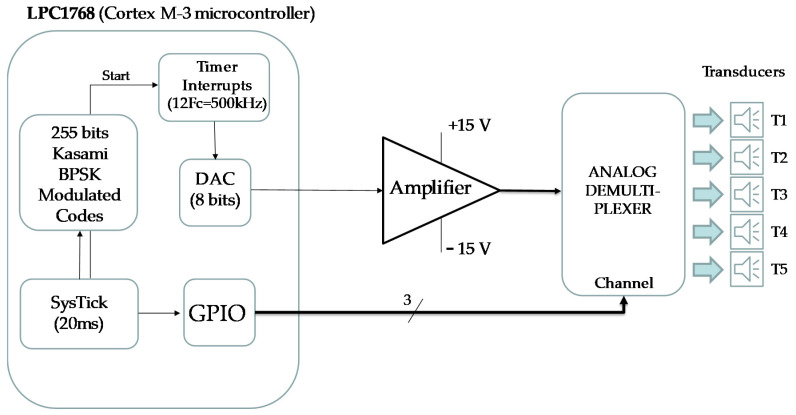
Block diagram of the circuit that implements the beaconing system with five emitters.

The electronic circuit that implements the beaconing system (see block diagram in Figure 14) is based on an NXP Cortex-M3 processor (LPC1768). The BPSK-modulated ultrasonic signal with the corresponding 1023-bit Kasami sequence applied to each transducer is obtained at the output of the internal DAC (Digital-Analog Converter), multiplexing over time the samples previously stored in memory for each of the five sequences assigned to beacons. The modulation symbol consists of 12 samples of a sine wave. The sampling frequency of the DAC is 500 kHz. The modulated signal is amplified before driving the transducer. The transducer used is the 328ST160 [32] at 41.667 kHz, where it has a linear behavior with the phase (see [23] for details).

The following are the experimental tests obtained from the ULPS, using the 4939 microphone as a receiver, with a preamplifier and a high-pass filter (15 kHz) stages, from Brüel-Kjaer [33]; and the commercial acquisition module Ultrasoundgate 116 Hm from Avisoft [34]. The sampling frequency used was 500 kHz.

**Figure 15:**
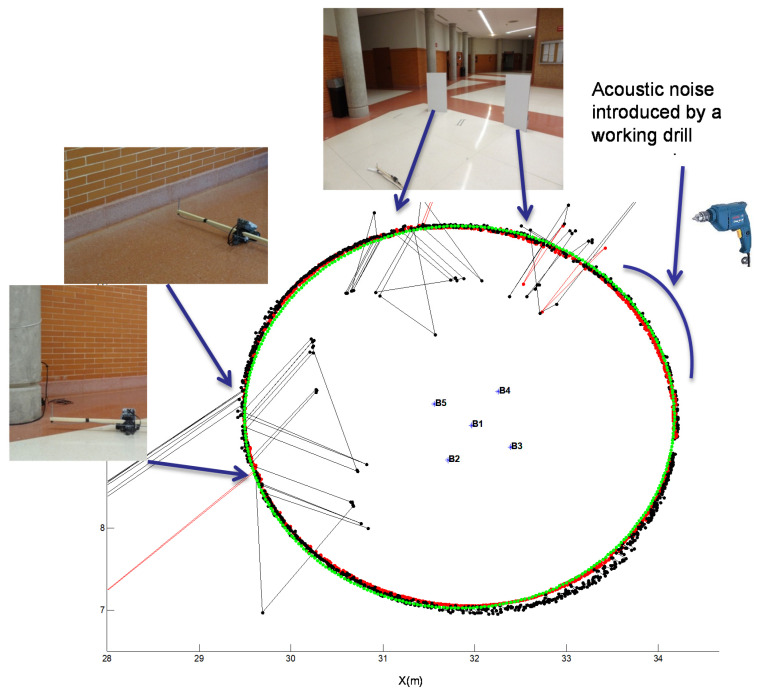
Circular path in the experimental environment with the designed ULPS. Real trajectory, in green; the estimated one using the GCC-PHAT(*β*) in red; and the estimated one using the CC in black. Different photos have been added with particular situations encountered by the receiver: high acoustic noise or multipath due to methacrylate plates, a wall or a column.

We performed a real experiment similar to the simulated one, using a Lego NXT robot that followed a circular path with a radius of 2.35 m and centered at the projection beacon 1 (reference beacon). The robot carried the microphone to continuously acquire the ultrasonic emissions. We chose a real environment where the multipath effect could appear. For that purpose, the robot navigated close to two columns and a wall. In addition, we placed two methacrylate plates next to the trajectory, working as reflectors to emphasize the multipath effect in other points of the path, as shown in Figure 15. In the last quadrant of the path, we manually introduced acoustic noise using a drill. Figure 15 clearly shows how the GCC-PHAT(*β*) (red) provides a better result compared to CC (black), by estimating the position practically at all the points marked on the path, thus making the ULPS more robust when the multipath effect appears.

**Figure 16:**
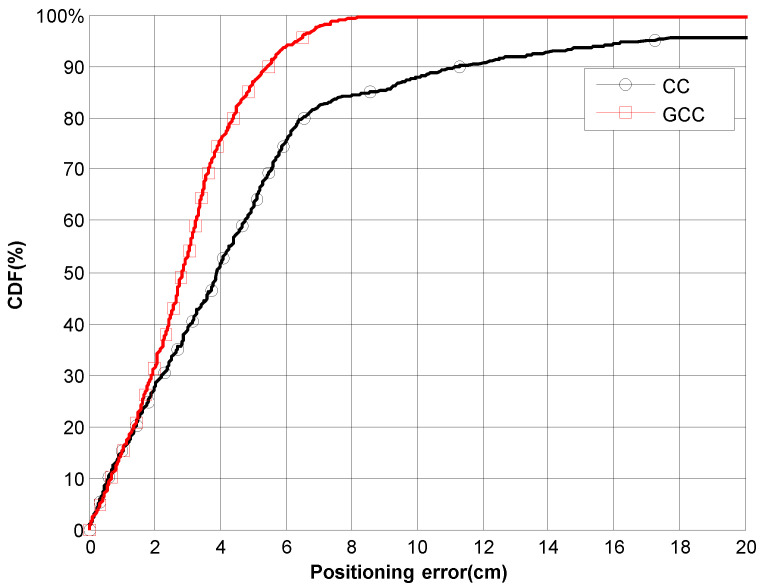
CDF of the ranging error in 2D positioning (CC vs. GCC-PHAT(*β*)) along the experimental path.

Figure 16 shows the CDF of the ranging error along the path. It can be observed that when using the GCC-PHAT(*β*), the position error is lower than 5.5 cm for 90% of measurements. In contrast, the CC results do not guarantee an error lower than 11.5 cm. The outliers due to the multipath effect are clearly noticeable in Figure 15 (those points far away from the real trajectory).

## 5. Conclusions

This work has proposed and validated an algorithm based on the use of the GCC-PHAT(*β*), for improving the detection of times-of-arrival in an ULPS. The performance of the detection of ToAs is improved whether the weighting factor of the PHAT filter is dynamically adjusted. After an empirical analysis, we derived a linear function to adjust the weighting factor, depending on the relationship between the maximum sidelobe and the main lobe of the GCC obtained after every measurement. We have tested this function through simulations that have proved the validity of the proposal in cases where the SNR is lower than 0 dB, or when the multipath effect hinders the detection of the ToAs. 

The algorithms have also been tested in a real ULPS based on five ultrasonic beacons that uses a mixed multiple medium access technique (T-CDMA), based on CDMA and TDMA). The receiver’s position is obtained by hyperbolic multilateration using TDoAs between a reference beacon and the other four, what avoids the use of synchronization between the beacons and the receiver. Experimental tests have been carried out with this ULPS to validate the proposal in a real environment, where the multipath effect appears and there are external sources of acoustic noise that make it difficult (or not possible) the signal detection with other methods.

Both simulated and experimental results show that the use of the proposed dynamic adjustment of the weighting factor in the GCC-PHAT provides a better accuracy of the positioning system in all the analyzed cases, compared with the standard CC. The proposal allows the achievement of positioning errors around 2 cm in most cases, even with multipath and high ambient noise.

## Figures and Tables

**Table 1:** Standard deviation (std in cm) of errors in 2D positioning with GCC-PHAT(*β*) for SNR = 20 dB, 0 dB and −10 dB.

	SNR = 20 dB		SNR = 0 dB		SNR = −10 dB		
*β*	Std X	Std Y	Std X	Std Y	Std X	Std Y	Outliers
1	0.77	0.55	2.81	0.47	31	26	30%
0.9	0.96	0.47	1.02	0.68	6.80	8.74	4%
0.8	0.87	0.52	1.05	0.65	8.74	9.79	4%
0.7	0.85	0.65	0.88	0.59	**0.94**	**0.61**	**2%**
0.6	0.92	0.57	0.95	0.72	2.28	2.15	6%
0.5	0.81	0.58	0.83	0.75	6.73	5.23	6%

## Data Availability

Not applicable.
